# Circulating IL-13 Is Associated with De Novo Development of HCC in HCV-Infected Patients Responding to Direct-Acting Antivirals

**DOI:** 10.3390/cancers12123820

**Published:** 2020-12-18

**Authors:** Zuzana Macek Jílková, Arnaud Seigneurin, Celine Coppard, Laurissa Ouaguia, Caroline Aspord, Patrice N. Marche, Vincent Leroy, Thomas Decaens

**Affiliations:** 1 Institute for Advanced Biosciences, Research Center UGA/Inserm U 1209/CNRS 5309, 38700 Grenoble, France; celine.coppard@hotmail.com (C.C.); louaguia@gpbscientific.com (L.O.); Caroline.Aspord@efs.sante.fr (C.A.); patrice.marche@univ-grenoble-alpes.fr (P.N.M.); vincent.leroy2@aphp.fr (V.L.); 2 Service d’Hépato-gastroentérologie, Pôle Digidune, CHU Grenoble Alpes, 38043 Grenoble, France; 3 University Grenoble Alpes, 38400 Saint-Martin-d’Hères, France; ASeigneurin@chu-grenoble.fr; 4 Service d’Evaluation Médicale, CHU Grenoble Alpes, 38043 Grenoble, France; 5 TIMC-IMAG UMR 5525, 38700 La Tronche, France; 6 Etablissement Français du Sang, Rhone-Alpes Auvergne, 38043 Grenoble, France

**Keywords:** hepatitis C virus, hepatocellular carcinoma, direct-acting antivirals, IL-13, IL-4, immune profile

## Abstract

**Simple Summary:**

Chronic hepatitis C virus infection is one of the major risk factors for the development of hepatocellular carcinoma. New direct-acting antivirals substantially improved the cure rate of hepatitis C, but the incidence of hepatitis C virus-related hepatocellular carcinoma remains high. To identify the immune profile associated with the risk for hepatocellular carcinoma, we investigated a cohort of patients who developed de novo hepatocellular carcinoma following direct-acting antiviral treatment in comparison to controls who did not develop hepatocellular carcinoma. Our results can improve clinical management prior to the development of hepatocellular carcinoma.

**Abstract:**

Direct-acting antivirals (DAAs) are highly effective in targeting hepatitis C virus (HCV) infections, but the incidence of HCV-related hepatocellular carcinoma (HCC) remains still high. In this study, we investigated a cohort of HCV-infected patients treated with DAAs who were followed up for 4 years after sustained virological response (SVR) achievement. Patients who developed de novo HCC following DAA treatment were compared to matched controls who did not develop HCC. These control patients were selected based on DAA treatment, sex, age, fibrosis status, and platelet counts. We evaluated serum levels of 30 immune mediators before, during, at the end of, and three months after DAA treatment using Luminex technology. We identified the immune factors associated with de novo HCC occurrence following DAA treatment. Specifically, interleukin (IL)-4 and IL-13 levels were significantly higher before start of the DAA treatment in the serum of patients who later developed HCC than in controls and stayed higher at each subsequent time point. Least absolute shrinkage and selection operator (LASSO) regression revealed IL-13 as the only strong factor associated with HCC development in this cohort of HCV patients. The difference was observed already at baseline of DAA treatment, which confirms the existence of a specific immune profile in these patients who later develop HCC.

## 1. Introduction

Chronic hepatitis C virus (HCV) infection is one of the major risk factors for hepatocellular carcinoma (HCC). The global prevalence of infection by HCV is estimated at 2.5% of the world population, and chronic state of the disease is associated with a 2–5% annual risk of developing HCC [1]. The introduction of direct-acting antivirals (DAAs) has dramatically changed the landscape of HCV therapy. DAAs are highly effective in targeting HCV infections, with a high rate of approximately 95% patients achieving a sustained virological response (SVR). This remarkable rate of DAA-induced SVR is associated with a reduction in HCC risk, but the incidence of HCV-related HCC remains still high, especially in patients with advanced fibrosis and cirrhosis [2,3,4]. Thus, circulating biomarkers and predictive factors for risk of development of HCC following DAA treatment are greatly needed to improve the clinical management of patients with chronic HCV prior to the development of HCC.

The liver immune system is modulated by HCV [5,6,7] and remains impaired even after the HCV infection heals [8]. The importance of the functional immune system in the defense against the development and progression of HCC is generally recognized. Debes et al. [9] showed immune-related differences in a small cohort of patients who developed HCC as either a recurrence or de novo following DAA treatment when compared to controls who did not develop HCC. These results suggested that individuals who developed HCC may have had a specific immune profile before the start of the DAA treatment.

In this study, we investigated a cohort of HCV-infected patients treated with DAAs who were followed up for 4 years after SVR achievement. Patients who developed de novo HCC following DAA treatment were compared to matched controls who did not develop HCC. Through a Luminex-based immunological analysis, we determined the modulation of immune profiles at four different time points—before, during, at the end of, and after DAA treatment—to identify putative factors associated with HCC development.

## 2. Results

Out of 334 patients, 13 patients developed de novo HCC following DAA treatment (corresponding to 3.9% of the entire cohort) with a median time of 14.9 months between start of the treatment and HCC diagnosis. Two patients who developed HCC shortly after DAA treatment were excluded from further analysis to avoid the impact of a possible preexisting HCC.

**Figure 1:**
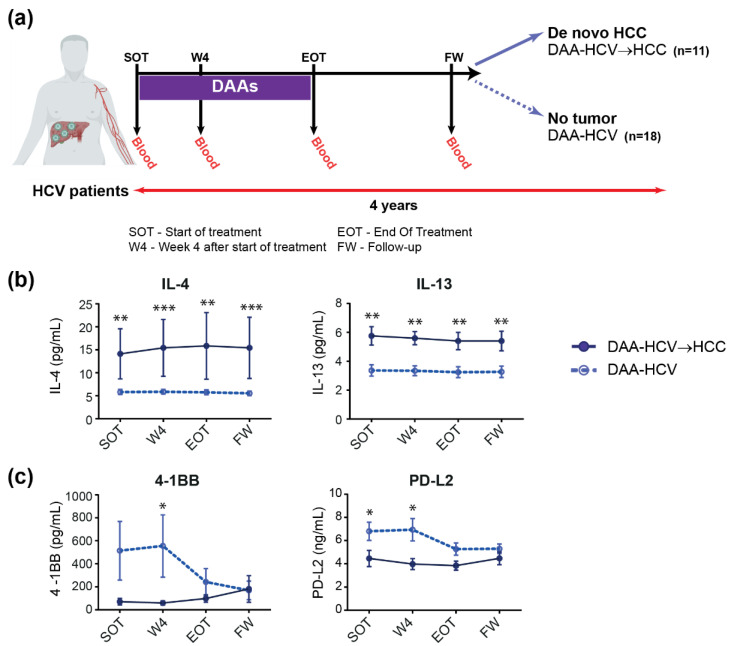
Design of study and circulating levels of immune mediators in hepatitis C virus (HCV)-infected patients treated by direct-acting antiviral (DAA). (**a**) Patients with chronic HCV were treated by DAA and followed up to check for hepatocellular carcinoma (HCC) development over a 4-year period (*n* = 334). Samples were collected at start of the treatment (SOT), four weeks after SOT (W4), end of the treatment (EOT), and 3 months after EOT as follow-up (FW). (**b**) Serum levels of interleukin (IL)-4 and IL-13 and (**c**) 4-1BB and programmed death (PD)-L2 in patients who developed de novo HCC following DAA treatment (DAA-HCV→HCC, *n* = 11) and patients who did not develop HCC (DAA-HCV, *n* = 18). * *p* < 0.05; ** *p* < 0.01; *** *p* < 0.001.

Eleven patient who developed HCC after DAA treatment were compared to eighteen matched controls who did not develop HCC during the follow-up of 4 years; Table 1, Figure 1a. Controls were selected based on DAA treatment, sex, age, fibrosis status, and platelet counts. The two classical markers of HCC (AFP and OPN) did not differ at the beginning of the treatment between DAA-treated patients who developed HCC and controls. Interestingly, we observed no differences in AFP levels even for each subsequent time point (Figure S1, Figure 1a). Similarly, the levels of liver enzymes GGT, ALT, and AST at baseline were the same, and no significant differences were observed in body weight, body mass index, history of diabetes mellitus, alcohol consumption, or tobacco smoking; Table 1.

Next, we measured immune mediators and selected those whose levels were significantly different in DAA-treated patients who developed HCC when compared with controls, as shown in Figure 1b,c. We observed that interleukin (IL)-4 and IL-13 levels were significantly higher before the start of the DAA treatment in serum from patients who later developed HCC than in controls (IL-4: 14.1 ± 5.5 vs. 5.8 ± 0.6 pg/mL, respectively, *p* = 0.0014; IL-13: 5.6 ± 0.6 vs. 3.4 ± 0.4 pg/mL, respectively, *p* = 0.0038) and stayed higher at each subsequent time point; Figure 1b. The circulating IL-4 area under the curve (AUC) value for receiver operating characteristic (ROC) curve analysis was 0.841 (0.687–0.995; *p* = 0.0024) and the IL-13 AUC value for ROC curve analysis was 0.813 (0.642–0.985; *p* = 0.0053) at baseline, revealing an effective discrimination capacity of these markers (Figure S1 and Figure 1b).

On the other hand, circulating serum levels of soluble 4-1BB and PD-L2 were lower in patients who later developed HCC than in controls; Figure 1c. Least absolute shrinkage and selection operator (LASSO) regression, using variables dichotomized according to the median value of HCC patients, selected IL-13 as the only strong factor associated with HCC development in HCV patients treated using DAA, at baseline, week 4, and at the end of treatment.

## 3. Discussion

Here, we identified immune factors associated with de novo HCC occurrence following DAA treatment. Circulating levels of IL-4 and IL-13 were significantly higher in patients who developed de novo HCC after DAA treatment compared to controls. The differences in cytokine levels were observed already at the baseline of DAA treatment, which confirms the existence of a specific immune profile in those patients who later developed HCC. LASSO regression revealed circulating IL-13 as the only strong factor associated with HCC development in this cohort of matched case-control HCV patients. Importantly, IL-4 and IL-13 are closely related to type 2 immune response-associated cytokines that induce the alternatively activated phenotype of macrophages [10,11,12] and are known to play a prominent role in promoting tumor progression [13]. A recent study demonstrated that IL-13 can induce an aggressive type of colorectal cancer by enhancing the expression of the epithelial–mesenchymal transition-promoting factor ZEB1 through the STAT6-dependent pathway [14]. Similarly, non-alcoholic fatty liver disease (NAFLD)-related HCC patients with cirrhosis have increased levels of circulating IL-13 compared to patients with cirrhosis but without HCC [15], and higher serum IL-13 levels were recently associated with HCC development in non-alcoholic steatohepatitis (NASH) [16]. Besides, high serum IL-4 levels were previously associated with poorer prognosis in patients with advanced HCC [17]. Thus, IL-4 and IL-13 cytokines are generally associated with carcinogenesis and may play a crucial role in immune-related mechanisms leading to the development of HCC.

We also observed a difference in levels of immune checkpoint 4-1BB, suggesting the possible defensive character of this immune mediator. This is in accordance with a recent study demonstrating that 4-1BB enhances CD8 T cell proliferation, survival, and effector functions of T cells in HCC [18]. However, a possible causal relationship between soluble 4-1BB or PD-L2 and anti-tumor defense still needs to be characterized.

Other potential predictive factors associated with HCC development after DAA treatment were previously determined [9,19]. Even though there are significant differences between the mentioned studies—mainly in terms of statistical analyses that have been used, selection of patients, and panel of studied markers—similarly to our study, Debes et al. identified increased circulating IL-4 and IL-13 levels in DAA-treated patients who later developed HCC when compared with controls. However, in Debes et al.’s study [9], patients who developed de novo HCC were mixed with patients who developed tumor recurrence, and relapse was observed in half of the patients who developed HCC while all patients in the control group achieved SVR. This difference in study design is likely the reason why our study did not confirm the other predictive markers for HCC development proposed by Debes et al. [9].

To investigate the immune profile associated with HCC development, we minimized the eventual impact of already known risk factors. Accordingly, patients with a history of HCC before DAA treatment and with the lack of SVR after DAA treatment were excluded from the analyses. Matched controls were selected based on treatment, sex, age, fibrosis status, platelet counts, and DAA treatment type. We believe that this approach is necessary to precisely investigate the existence of an immune profile related to de novo HCC development. However, it should be mentioned that other factors may also contribute to higher risk of HCC development, such as a family history of HCC [20] or NAFLD/NASH risk factors [21].

Even though the present study does not investigate the mechanisms linking IL-4 and IL-13 levels to de novo HCC development after DAA treatment, our results help to understand the immune modifications associated with HCC occurrence. Confirmation of IL-4 and IL-13 commitment using a larger and independent cohort is mandatory.

## 4. Materials and Methods

### 4.1. Patient Population

Patients with chronic HCV who enrolled at the Department of Hepatology and Gastroenterology, Centre Hospitalier Universitaire (CHU) Grenoble-Alpes, between 2014 and 2015 were treated using DAA and followed up to check for HCC development over a 4-year period (*n* = 334). After the patients had consented, blood was drawn from them and the serum was processed according to standard protocols. Samples were collected at 4 different time points: (i) start of the treatment (SOT), (ii) four weeks after SOT (W4), (iii) end of the treatment (EOT), and (iv) 3 months after EOT as follow-up (FW), as shown in Figure 1a. The sample collection was reviewed and approved by the ethics committee of CHU Grenoble: AC-2014-2094 #3 (DC-2008-727). Patients with a history of HCC before DAA treatment and with a lack of SVR after DAA treatment were excluded from future analyses. Thirteen patients developed de novo HCC after DAA treatment. To avoid a possible preexisting HCC, two patients were excluded from study as they developed HCC very shortly after DAA treatment. The study was designed as a case-control study. Matched controls were selected based on DAA treatment, sex, age, fibrosis status, and platelet counts. All patients were subjected to liver stiffness assessment by Fibroscan^®^, and moreover, in several cases, liver biopsy was also performed and used for histological examination. Liver fibrosis was staged on a 0–4 scale according to the Metavir scoring system. The patients’ characteristics are summarized in Table 1.

### 4.2. Multiplex Assay

Circulating immune mediators were quantified by Luminex MAGPIX (Thermo Fisher Scientific, Waltham, MA, USA) according to the manufacturer’s instructions. The following checkpoint molecules and cytokines were measured: Programmed death (PD)-1, PD-L1, PD-L2, CD152 (CTLA-4), TIM-3, GITR, GITRL, LAG-3, CD137 (4-1BB), IL-6, TNF-alpha, IFN-gamma, IL-10, IL-12p70, IL-1 beta, IL-4, IL-5, IL-17A, IL-13, IL-29, IL-22, TRAIL, MICB, MICA, IDO, Perforin, IL-2, CD30, VEGF, APRIL, TGF-beta. Data were analyzed using ProcartaPlexAnalyst 1.0 (Thermo Fisher Scientific, Waltham, MA, USA).

### 4.3. Enzyme-Linked Immunosorbent Assay (ELISA)

Serum samples were analyzed to determine the levels of alpha fetoprotein (AFP) and osteopontin (OPN) by human alpha-fetoprotein Duo Set Elisa (R&D Systems, DY1369, Minneapolis, MN 55413, USA) and human osteopontin Duo set Elisa (R&D Systems, DY1433, Minneapolis, MN 55413, USA).

### 4.4. Statistical Analysis

Analyses were performed using the statistical software GraphPad Prism 6 (GraphPad Software, San Diego, CA, USA). Normal distribution was tested by the D’Agostino–Pearson omnibus normality test. When data derived from both cohorts were normally distributed, the unpaired t-test was used to determine significant differences observed between the groups. On the other hand, when data from either cohort were not normally distributed, the Mann–Whitney test was performed. A LASSO logistic regression was used to select the factors associated with HCC development. R software (version 3.6.1) by the R Foundation for Statistical Computing (Vienna, Austria) was used to perform the LASSO model.

## 5. Conclusions

We identified immune factors associated with de novo HCC occurrence following DAA treatment. Differences in cytokine levels were observed already at the baseline of DAA treatment, which confirms the existence of a specific immune profile in those patients who later developed HCC compared with controls.

## Figures and Tables

**Table 1:** Characteristics of HCV-infected patients treated by DAA.

At Start of Treatment	DAA-HCV→HCC (*n* = 11)	DAA-HCV (*n* = 18)	*p* Value
Age (years)	57.6 ± 5.6	57.2 ± 4.8	0.8475
Sex (number, % men)	11, 100%	18, 100%	>0.999
Fibrosis stage F3–F4/F4	0/11	2/16	0.5123
Platelet count *	113 ± 50	121 ± 48	0.6487
AFP (ng/mL) *	14.7 ± 9.3; 11.5 (4–36)	20.8 ± 20; 13.8 (7–94)	0.3795
OPN (ng/mL) *	60.9 ± 51.9; 54 (24–211)	47.5 ± 20.7; 44 (19–103)	0.7993
GGT (UL/mL) *	200 ± 158; 151 (81–573)	237 ± 207; 118 (60–646)	0.5864
AST (UL/mL) *	112 ± 72; 101 (50–314)	113 ± 58; 95 (31–236)	0.8251
ALT (UL/mL) *	149 ± 150; 86 (50–577)	124 ± 88; 100 (40–346)	0.7996
Body weight (kg)	74.2 ± 9.8	80.1 ± 13.7	0.2241
BMI	24.5 ± 2.7	27.2 ± 4.5	0.0818
History of diabetes (number, %)	2, 18.2%	4, 22.2%	>0.999
Alcohol consumption (number, %)	7, 63.6%	9, 50%	0.2430
Tobacco smoking (number, %)	7, 63.6%	11, 61.1%	>0.999
HCV Genotype 1/3/4	6/4/1	11/6/1	>0.852
HBV co-infection (number, %)	6, 54.5%	55.6%	>0.959
Treatment outcome			
SVR (number, %)	11, 100%	18, 100%	>0.999
HCC development			
Start of DAA treatment to HCC (months) *	19.9 ± 10.3; 15.8 [13.6–40.5]	-	
Number of tumor 1/2	8/3	-	
Size of biggest tumor (mm) *	24.2 ± 13.6; 18 [12–52]	-	

Patients who developed de novo hepatocellular carcinoma (HCC) following direct-acting antivirals (DAA) treatment (DAA-HCV→HCC) were compared to matched controls who did not develop HCC (DAA-HCV). Alpha Fetoprotein, AFP; Osteopontin, OPN; Gamma-glutamyltransferase, GGT; Aspartate aminotransferase, AST; Alanine aminotransferase, ALT; Sustained virologic response, SVR; Body mass index, BMI. Data normally distributed, Mean ± SD, unpaired *t*-test. * Data not normally distributed, Mean ± SD; Median (Min–Max), Mann–Whitney test.

## Supplementary Materials

The following are available online at https://www.mdpi.com/2072-6694/12/12/3820/s1, Figure S1: Serum levels of AFP and receiver operating characteristic (ROC) curve analysis.
