# Supplementary material for: Circulating IL-13 Is Associated with De Novo Development of HCC in HCV-Infected Patients Responding to Direct-Acting Antivirals
Source: Cancers (Basel). 2020 Dec 18;12(12):3820. doi: 10.3390/cancers12123820 (PMC7766760; doi:10.3390/cancers12123820)
Supplement: Supplementary file 1 [file cancers-12-03820-s001.pdf]

cancers

Supplementary Materials

Circulating IL-13 is associated with de novo development of HCC in HCV-infected patients responding to direct-acting antivirals

Zuzana Macek Jílková, Arnaud Seigneurin, Celine Coppard, Laurissa Ouaguia, Caroline Aspord, Patrice N. Marche, Vincent Leroy and Thomas Decaens

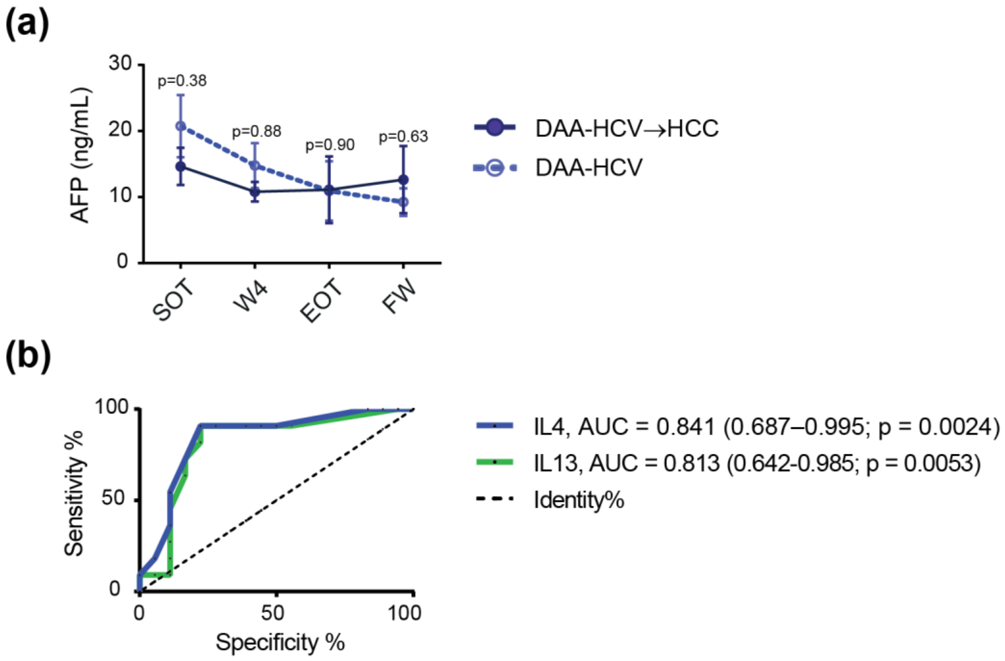

Figure S1. Serum levels of AFP and receiver operating characteristic (ROC) curve analysis. **a**) Serum levels of AFP in patients who developed de-novo HCC following DAA treatment (DAA-HCV→HCC, n=11) and patients who did not develop HCC (DAA-HCV, n=18). Samples were collected at start of the treatment (SOT), four weeks after SOT (W4), end of the treatment (EOT), and 3 months after EOT as follow-up (FW). **b**) IL-4 area under the curve (AUC) values for ROC curve analysis were 0.841 (0.687–0.995; p = 0.0024) and IL-13 AUC values for ROC curve analysis were 0.813 (0.642-0.985; p = 0.0053) at baseline, revealing an effective discrimination capacity of these markers.
